# *catena*-Poly[[[(1-ethyl-6-fluoro-4-oxo-7-(piperazin-1-yl)-1,4-dihydro­quinoline-3-carboxyl­ato-κ^2^ *O* ^3^,*O* ^4^)copper(II)]-μ-1-ethyl-6-fluoro-4-oxo-7-(piperazin-1-yl)-1,4-dihydro­quinoline-3-carboxyl­ato-κ^3^ *N* ^7′^:*O* ^3^,*O* ^4^] tetra­hydrate]

**DOI:** 10.1107/S1600536808026469

**Published:** 2008-08-23

**Authors:** Shu-Ye Wang, Xue-Ming Song, Li-Xiang Duan, Zhe An

**Affiliations:** a The First Affiliated Hospital, Harbin Medical University, Harbin, People’s Republic of China; b School of Chemistry and Life Science, Maoming University, Maoming 525000, People’s Republic of China

## Abstract

In the title compound, {[Cu(C_16_H_17_FN_3_O_3_)_2_]·4H_2_O}_*n*_, the Cu^II^ atom is bonded to two *O*,*O*′-bidentate 1-ethyl-6-fluoro-4-oxo-7-(piperazin-1-yl)-1,4-dihydro­quinoline-3-carboxyl­ate (norf) monoanions and a symmetry-generated *N*-bonded norf anion, resulting in a distorted square-pyramidal coordination environ­ment with the N atom occupying the apical site. The bridging norf anion results in one-dimensional chains propogating along [010]. A network of O—H⋯O and N—H⋯O hydrogen bonds helps to establish the crystal structure.

## Related literature

For the iron, zinc and cobalt complexes of the norf anion, see: Chen *et al.* (2001 ▶); Qu *et al.* (2003 ▶); An *et al.* (2007 ▶). For background on the medicinal uses of Hnorf, see: Mizuki *et al.* (1996 ▶).
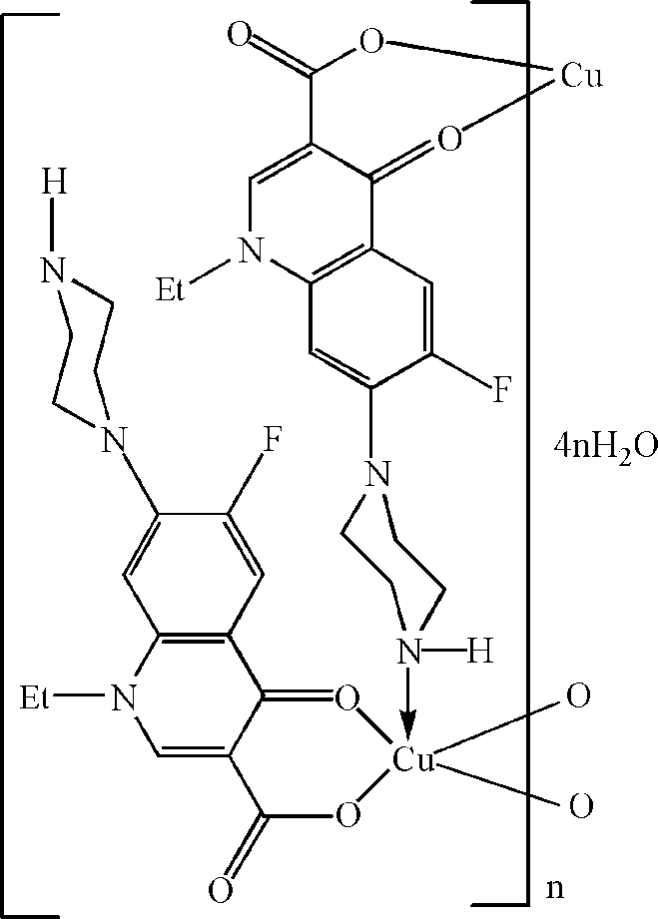


## Experimental

### 

#### Crystal data

[Cu(C_16_H_17_FN_3_O_3_)_2_]·4H_2_O
*M* *_r_* = 772.26
Triclinic, 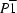

*a* = 10.023 (2) Å
*b* = 11.708 (2) Å
*c* = 16.219 (3) Å
α = 97.22 (3)°
β = 107.05 (3)°
γ = 105.00 (3)°
*V* = 1715.2 (6) Å^3^
*Z* = 2
Mo *K*α radiation
μ = 0.71 mm^−1^
*T* = 296 (2) K
0.43 × 0.28 × 0.22 mm

#### Data collection

Bruker APEXII CCD diffractometer
Absorption correction: multi-scan (*SADABS*; Bruker, 2004 ▶) *T*_min_ = 0.749, *T*_max_ = 0.859
13404 measured reflections
5960 independent reflections
4481 reflections with *I* > 2σ(*I*)
*R*_int_ = 0.028

#### Refinement

*R*[*F* ^2^ > 2σ(*F* ^2^)] = 0.054
*wR*(*F* ^2^) = 0.191
*S* = 1.00
5960 reflections
492 parameters
H atoms treated by a mixture of independent and constrained refinement
Δρ_max_ = 1.90 e Å^−3^
Δρ_min_ = −0.72 e Å^−3^

### 

Data collection: *APEX2* (Bruker, 2004 ▶); cell refinement: *SAINT-Plus* (Bruker, 2004 ▶); data reduction: *SAINT-Plus*; program(s) used to solve structure: *SHELXS97* (Sheldrick, 2008 ▶); program(s) used to refine structure: *SHELXL97* (Sheldrick, 2008 ▶); molecular graphics: *SHELXTL* (Sheldrick, 2008 ▶); software used to prepare material for publication: *SHELXTL*.

## Supplementary Material

Crystal structure: contains datablocks I, global. DOI: 10.1107/S1600536808026469/hb2782sup1.cif

Structure factors: contains datablocks I. DOI: 10.1107/S1600536808026469/hb2782Isup2.hkl

Additional supplementary materials:  crystallographic information; 3D view; checkCIF report

## Figures and Tables

**Table 1:** Selected bond lengths (Å)

Cu1—O3	1.936 (3)
Cu1—O1	1.939 (3)
Cu1—O4	1.944 (3)
Cu1—O5	1.953 (3)
Cu1—N6^i^	2.248 (3)

Symmetry code: (i) 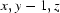.

**Table 2:** Hydrogen-bond geometry (Å, °)

*D*—H⋯*A*	*D*—H	H⋯*A*	*D*⋯*A*	*D*—H⋯*A*
O1*W*—H1*W*⋯O4*W*	0.83 (9)	2.08 (10)	2.735 (14)	136 (10)
O1*W*—H2*W*⋯O3*W*^ii^	0.83 (8)	1.78 (8)	2.580 (11)	163 (11)
O3*W*—H5*W*⋯O1*W*^i^	0.88 (6)	2.17 (11)	2.580 (11)	108 (9)
O3*W*—H6*W*⋯O1	0.83 (8)	2.13 (8)	2.865 (8)	147 (9)
O4*W*—H8*W*⋯O1*W*	0.90 (9)	2.17 (11)	2.735 (14)	121 (10)
N6—H6*A*⋯O1*W*^iii^	1.05 (3)	2.12 (4)	3.105 (12)	155 (3)

Symmetry codes: (i) 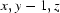; (ii) 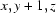; (iii) 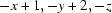.

**Fig. 1.**
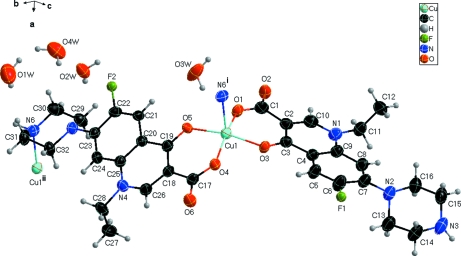
The asymmetric unit of (I), expanded to show the coordination of the Cu atom, drawn with 30% probability displacement ellipsoids for the non-hydrogen atoms (arbitrary spheres for the H atoms). Symmetry code: (i) x, 1-y, z.

**Fig. 2.**
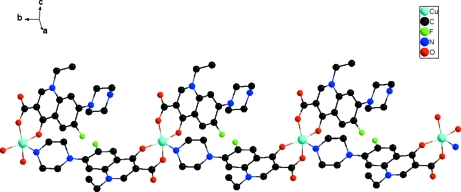
Part of the chain structure of (I).

## supplementary crystallographic information

### Comment

Norfloxacin (*H*-Norf,1-ethyl-6-fluoro-1,4-dihydro-4-oxo-7- (1-piperazinyl)-3-quinoline carboxylic acid) is member of the class of quinolones that is used to treat infections (Mizuki *et al.* 1996). The zinc(II), iron(II) and cobalt(II) derivatives of norf have been reported (Chen. *et al.*, 2001; Qu *et al.* 2003; An *et al.*, 2007).

The title copper(II) derivative, (I), a one-dimensional coordination polymer in which the anion acts in a bridging mode, is reported here (Fig. 1).

The Cu(II) atom is coordinated (Table 1) by four oxygen atoms and one N atoms from three norfloxacin ligands (one monodentate-N and two O,*O*-bidentate) to form a one-dimensional coordination polymer (Fig. 2).  A network of O—H···O and N—H···O hydrogen bonds (Table 2) helps to establish the packing.

### Experimental

A mixture of Cu(CH_3_COO)_2_.H_2_O (0.05 g, 0.25 mmol), Hnorf( 0.16 g, 0.50 mmol), sodium hydroxide (0.02 g, 0.50 mmol) and water (12 ml) was stirred for 20 min in air. The mixture was then transferred to a 23 ml Teflon-lined hydrothermal bomb. The bomb was kept at 443 K for 96 h under autogenous pressure. After cooling, blue blocks of (I) were obtained from the reaction mixture.

### Refinement

The N- and O-bonded H atoms were located in difference maps and their positions were freely refined with a fixed U_iso_ value.  This has led to some very short H···H intermolecular contacts and the positions of these H atoms should be regarded as less certain.

All the C-bonded H atoms were placed in calculated positions with C—H = 0.93Å and refined as riding with *U*_iso_(H) = 1.2*U*_eq_(carrier).

### Crystal data

**Table:**

[Cu(C_16_H_17_FN_3_O_3_)_2_]·4H_2_O	*Z* = 2
*M**_r_* = 772.26	*F*_000_ = 806
Triclinic, *P*1	*D*_x_ = 1.495 Mg m^−^^3^
Hall symbol: -P 1	Mo *K*α radiation λ = 0.71073 Å
*a* = 10.023 (2) Å	Cell parameters from 5960 reflections
*b* = 11.708 (2) Å	θ = 3.1–25.1º
*c* = 16.219 (3) Å	µ = 0.71 mm^−^^1^
α = 97.22 (3)º	*T* = 296 (2) K
β = 107.05 (3)º	Block, blue
γ = 105.00 (3)º	0.43 × 0.28 × 0.22 mm
*V* = 1715.2 (6) Å^3^	

### Data collection

**Table:**

Bruker APEXII CCD diffractometer	5960 independent reflections
Radiation source: fine-focus sealed tube	4481 reflections with *I* > 2σ(*I*)
Monochromator: graphite	*R*_int_ = 0.028
*T* = 296(2) K	θ_max_ = 25.1º
ω scans	θ_min_ = 3.1º
Absorption correction: multi-scan(SADABS; Bruker, 2004)	*h* = −11→11
*T*_min_ = 0.749, *T*_max_ = 0.859	*k* = −13→13
13404 measured reflections	*l* = −19→19

### Refinement

**Table:**

Refinement on *F*^2^	Secondary atom site location: difference Fourier map
Least-squares matrix: full	Hydrogen site location: difmap and geom
*R*[*F*^2^ > 2σ(*F*^2^)] = 0.054	H atoms treated by a mixture of independent and constrained refinement
*wR*(*F*^2^) = 0.191	*w* = 1/[σ^2^(*F*_o_^2^) + (0.136*P*)^2^ + 1.0468*P*] where *P* = (*F*_o_^2^ + 2*F*_c_^2^)/3
*S* = 1.00	(Δ/σ)_max_ < 0.001
5960 reflections	Δρ_max_ = 1.90 e Å^−^^3^
492 parameters	Δρ_min_ = −0.72 e Å^−^^3^
Primary atom site location: structure-invariant direct methods	Extinction correction: none

### Special details

**Table:**

Geometry. All e.s.d.'s (except the e.s.d. in the dihedral angle between two l.s. planes) are estimated using the full covariance matrix. The cell e.s.d.'s are taken into account individually in the estimation of e.s.d.'s in distances, angles and torsion angles; correlations between e.s.d.'s in cell parameters are only used when they are defined by crystal symmetry. An approximate (isotropic) treatment of cell e.s.d.'s is used for estimating e.s.d.'s involving l.s. planes.
Refinement. Refinement of *F*^2^ against ALL reflections. The weighted *R*-factor *wR* and goodness of fit *S* are based on *F*^2^, conventional *R*-factors *R* are based on *F*, with *F* set to zero for negative *F*^2^. The threshold expression of *F*^2^ > σ(*F*^2^) is used only for calculating *R*-factors(gt) *etc*. and is not relevant to the choice of reflections for refinement. *R*-factors based on *F*^2^ are statistically about twice as large as those based on *F*, and *R*- factors based on ALL data will be even larger.

### Fractional atomic coordinates and isotropic or equivalent isotropic displacement parameters (Å^2^)

**Table:**

	*x*	*y*	*z*	*U*_iso_*/*U*_eq_	
Cu1	0.90729 (5)	0.04270 (4)	0.21869 (3)	0.0338 (2)	
C1	0.6975 (4)	0.0019 (4)	0.3152 (3)	0.0439 (10)	
C2	0.7856 (4)	−0.0629 (3)	0.3706 (3)	0.0373 (9)	
C3	0.9119 (4)	−0.0878 (3)	0.3593 (3)	0.0342 (8)	
C4	0.9788 (4)	−0.1560 (3)	0.4170 (3)	0.0362 (9)	
C5	1.0984 (5)	−0.1904 (4)	0.4073 (3)	0.0436 (10)	
H5	1.1368	−0.1669	0.3642	0.052*	
C6	1.1580 (5)	−0.2569 (5)	0.4601 (3)	0.0524 (11)	
C7	1.1120 (5)	−0.2918 (4)	0.5307 (3)	0.0442 (10)	
C8	0.9952 (5)	−0.2557 (4)	0.5407 (3)	0.0408 (9)	
H8	0.9617	−0.2748	0.5866	0.049*	
C9	0.9262 (4)	−0.1917 (4)	0.4842 (3)	0.0377 (9)	
C10	0.7405 (4)	−0.1000 (4)	0.4376 (3)	0.0424 (10)	
H10	0.6590	−0.0819	0.4448	0.051*	
C11	0.7415 (5)	−0.1957 (5)	0.5614 (3)	0.0532 (12)	
H11A	0.6805	−0.1459	0.5693	0.064*	
H11B	0.8203	−0.1801	0.6172	0.064*	
C12	0.6511 (7)	−0.3266 (6)	0.5376 (5)	0.0801 (18)	
H12A	0.5717	−0.3422	0.4830	0.120*	
H12B	0.6122	−0.3452	0.5835	0.120*	
H12C	0.7115	−0.3763	0.5309	0.120*	
C13	1.3388 (5)	−0.3119 (5)	0.6269 (4)	0.0571 (12)	
H13A	1.3626	−0.2425	0.6742	0.068*	
H13B	1.3845	−0.2851	0.5847	0.068*	
C14	1.3980 (6)	−0.4084 (5)	0.6642 (3)	0.0574 (12)	
H14A	1.3819	−0.4739	0.6157	0.069*	
H14B	1.5031	−0.3738	0.6944	0.069*	
C15	1.1717 (6)	−0.5018 (5)	0.6819 (4)	0.0594 (13)	
H15A	1.1267	−0.5291	0.7244	0.071*	
H15B	1.1474	−0.5715	0.6348	0.071*	
C16	1.1055 (6)	−0.4098 (4)	0.6437 (3)	0.0505 (11)	
H16A	1.0011	−0.4476	0.6124	0.061*	
H16B	1.1180	−0.3442	0.6910	0.061*	
C17	1.1846 (4)	0.1672 (4)	0.1965 (3)	0.0349 (9)	
C18	1.1248 (4)	0.2682 (3)	0.1721 (2)	0.0320 (8)	
C19	0.9742 (4)	0.2613 (3)	0.1532 (3)	0.0327 (8)	
C20	0.9356 (4)	0.3645 (3)	0.1262 (3)	0.0319 (8)	
C21	0.7879 (4)	0.3613 (3)	0.1007 (3)	0.0338 (9)	
H21	0.7149	0.2912	0.0973	0.041*	
C22	0.7527 (4)	0.4610 (3)	0.0811 (3)	0.0362 (9)	
C23	0.8575 (4)	0.5732 (3)	0.0900 (3)	0.0333 (8)	
C24	1.0026 (4)	0.5748 (3)	0.1113 (3)	0.0351 (9)	
H24	1.0749	0.6456	0.1152	0.042*	
C25	1.0414 (4)	0.4703 (3)	0.1269 (3)	0.0331 (8)	
C26	1.2212 (4)	0.3712 (4)	0.1661 (3)	0.0383 (9)	
H26	1.3180	0.3726	0.1767	0.046*	
C27	1.3780 (7)	0.6550 (5)	0.2444 (5)	0.0809 (19)	
H27A	1.3050	0.6809	0.2618	0.121*	
H27B	1.4552	0.7247	0.2459	0.121*	
H27C	1.4179	0.6088	0.2844	0.121*	
C28	1.3088 (5)	0.5775 (4)	0.1518 (4)	0.0493 (11)	
H28A	1.2717	0.6252	0.1112	0.059*	
H28B	1.3826	0.5511	0.1344	0.059*	
C29	0.7251 (5)	0.7081 (4)	0.1276 (3)	0.0423 (10)	
H29A	0.7885	0.7424	0.1885	0.051*	
H29B	0.6495	0.6362	0.1265	0.051*	
C30	0.6551 (5)	0.7987 (4)	0.0915 (4)	0.0526 (12)	
H30A	0.5873	0.7627	0.0317	0.063*	
H30B	0.6001	0.8212	0.1275	0.063*	
C31	0.8498 (5)	0.8720 (4)	0.0359 (3)	0.0469 (11)	
H31A	0.9240	0.9430	0.0344	0.056*	
H31B	0.7829	0.8360	−0.0240	0.056*	
C32	0.9225 (5)	0.7818 (3)	0.0719 (3)	0.0387 (9)	
H32A	0.9756	0.7589	0.0348	0.046*	
H32B	0.9923	0.8188	0.1310	0.046*	
F1	1.2644 (4)	−0.2977 (4)	0.4433 (2)	0.0794 (11)	
F2	0.6077 (2)	0.4524 (2)	0.05141 (19)	0.0529 (7)	
N1	0.8050 (4)	−0.1605 (3)	0.4935 (2)	0.0412 (8)	
N2	1.1767 (4)	−0.3611 (4)	0.5827 (3)	0.0474 (9)	
N3	1.3301 (5)	−0.4575 (4)	0.7252 (3)	0.0611 (11)	
H3B	1.3751 (17)	−0.441 (4)	0.7842 (7)	0.073*	
N4	1.1867 (3)	0.4699 (3)	0.1462 (2)	0.0366 (7)	
N5	0.8124 (4)	0.6743 (3)	0.0745 (2)	0.0372 (8)	
N6	0.7690 (4)	0.9081 (3)	0.0911 (2)	0.0392 (8)	
H6A	0.723 (3)	0.9747 (16)	0.069 (3)	0.047*	
O1	0.7412 (3)	0.0469 (3)	0.2558 (2)	0.0474 (7)	
O2	0.5865 (4)	0.0120 (4)	0.3291 (3)	0.0752 (12)	
O3	0.9694 (3)	−0.0539 (3)	0.3021 (2)	0.0424 (7)	
O4	1.1027 (3)	0.0746 (2)	0.2105 (2)	0.0403 (7)	
O5	0.8753 (3)	0.1742 (2)	0.1603 (2)	0.0419 (7)	
O6	1.3139 (3)	0.1796 (3)	0.2032 (3)	0.0584 (9)	
O1W	0.4376 (13)	0.9606 (8)	0.0102 (6)	0.182 (4)	
O2W	0.2803 (6)	0.9249 (5)	0.2713 (5)	0.122 (2)	
O3W	0.5350 (6)	0.1403 (7)	0.1444 (4)	0.109 (2)	
O4W	0.2705 (8)	0.8303 (7)	0.0935 (7)	0.143 (3)	
H1W	0.365 (9)	0.903 (8)	0.006 (7)	0.171*	
H2W	0.473 (12)	1.008 (9)	0.059 (4)	0.171*	
H3W	0.309 (13)	0.974 (7)	0.319 (4)	0.171*	
H4W	0.240 (11)	0.855 (3)	0.274 (6)	0.171*	
H5W	0.472 (9)	0.069 (4)	0.138 (8)	0.171*	
H6W	0.615 (5)	0.143 (8)	0.180 (6)	0.171*	
H7W	0.351 (6)	0.819 (10)	0.100 (9)	0.171*	
H8W	0.265 (11)	0.888 (6)	0.062 (8)	0.171*	

### Atomic displacement parameters (Å^2^)

**Table:**

	*U*^11^	*U*^22^	*U*^33^	*U*^12^	*U*^13^	*U*^23^
Cu1	0.0306 (3)	0.0301 (3)	0.0431 (3)	0.0152 (2)	0.0094 (2)	0.0124 (2)
C1	0.030 (2)	0.045 (2)	0.061 (3)	0.0190 (18)	0.0139 (19)	0.016 (2)
C2	0.0310 (19)	0.0345 (19)	0.046 (2)	0.0103 (16)	0.0122 (17)	0.0070 (17)
C3	0.0313 (18)	0.0358 (19)	0.035 (2)	0.0104 (16)	0.0111 (16)	0.0074 (16)
C4	0.0335 (19)	0.0365 (19)	0.037 (2)	0.0114 (17)	0.0093 (16)	0.0087 (16)
C5	0.044 (2)	0.059 (3)	0.041 (2)	0.029 (2)	0.0185 (18)	0.022 (2)
C6	0.055 (3)	0.071 (3)	0.049 (3)	0.037 (2)	0.024 (2)	0.026 (2)
C7	0.045 (2)	0.054 (2)	0.037 (2)	0.021 (2)	0.0110 (18)	0.0162 (19)
C8	0.044 (2)	0.046 (2)	0.033 (2)	0.0144 (19)	0.0133 (17)	0.0109 (17)
C9	0.038 (2)	0.041 (2)	0.033 (2)	0.0136 (18)	0.0114 (16)	0.0066 (16)
C10	0.036 (2)	0.043 (2)	0.051 (3)	0.0161 (18)	0.0178 (19)	0.0069 (19)
C11	0.055 (3)	0.076 (3)	0.043 (3)	0.032 (3)	0.026 (2)	0.021 (2)
C12	0.066 (3)	0.095 (4)	0.082 (4)	0.009 (3)	0.036 (3)	0.034 (4)
C13	0.054 (3)	0.068 (3)	0.055 (3)	0.027 (2)	0.014 (2)	0.028 (2)
C14	0.061 (3)	0.074 (3)	0.044 (3)	0.038 (3)	0.012 (2)	0.020 (2)
C15	0.077 (3)	0.050 (3)	0.051 (3)	0.020 (3)	0.017 (2)	0.024 (2)
C16	0.061 (3)	0.054 (3)	0.043 (3)	0.021 (2)	0.020 (2)	0.020 (2)
C17	0.0322 (19)	0.040 (2)	0.038 (2)	0.0190 (17)	0.0122 (16)	0.0118 (16)
C18	0.0323 (18)	0.0334 (18)	0.032 (2)	0.0159 (16)	0.0085 (15)	0.0082 (15)
C19	0.0359 (19)	0.0289 (18)	0.035 (2)	0.0141 (16)	0.0102 (16)	0.0081 (15)
C20	0.0317 (18)	0.0259 (17)	0.035 (2)	0.0099 (15)	0.0067 (15)	0.0056 (15)
C21	0.0264 (17)	0.0283 (17)	0.041 (2)	0.0076 (15)	0.0035 (15)	0.0086 (15)
C22	0.0276 (18)	0.0297 (18)	0.047 (2)	0.0131 (16)	0.0036 (16)	0.0061 (16)
C23	0.0366 (19)	0.0266 (17)	0.034 (2)	0.0124 (16)	0.0062 (16)	0.0065 (15)
C24	0.036 (2)	0.0296 (18)	0.039 (2)	0.0080 (16)	0.0124 (16)	0.0115 (16)
C25	0.0346 (19)	0.0308 (18)	0.033 (2)	0.0119 (16)	0.0090 (15)	0.0082 (15)
C26	0.0303 (19)	0.044 (2)	0.045 (2)	0.0176 (17)	0.0122 (17)	0.0162 (18)
C27	0.062 (3)	0.056 (3)	0.092 (5)	−0.004 (3)	−0.002 (3)	0.017 (3)
C28	0.036 (2)	0.043 (2)	0.075 (3)	0.0081 (19)	0.027 (2)	0.024 (2)
C29	0.040 (2)	0.0281 (18)	0.063 (3)	0.0105 (17)	0.023 (2)	0.0081 (18)
C30	0.040 (2)	0.029 (2)	0.078 (4)	0.0104 (19)	0.009 (2)	0.000 (2)
C31	0.069 (3)	0.0309 (19)	0.042 (2)	0.018 (2)	0.015 (2)	0.0146 (17)
C32	0.047 (2)	0.0285 (18)	0.047 (2)	0.0160 (17)	0.0207 (19)	0.0135 (17)
F1	0.090 (2)	0.135 (3)	0.077 (2)	0.090 (2)	0.0545 (19)	0.067 (2)
F2	0.0305 (12)	0.0325 (12)	0.0806 (19)	0.0100 (10)	−0.0026 (11)	0.0106 (11)
N1	0.0374 (17)	0.0491 (19)	0.040 (2)	0.0172 (16)	0.0145 (15)	0.0094 (16)
N2	0.051 (2)	0.056 (2)	0.042 (2)	0.0260 (18)	0.0144 (16)	0.0198 (17)
N3	0.079 (3)	0.065 (3)	0.042 (2)	0.034 (2)	0.009 (2)	0.022 (2)
N4	0.0315 (16)	0.0362 (17)	0.046 (2)	0.0113 (14)	0.0156 (14)	0.0152 (14)
N5	0.0391 (17)	0.0275 (15)	0.046 (2)	0.0146 (14)	0.0110 (15)	0.0127 (14)
N6	0.0406 (18)	0.0275 (15)	0.045 (2)	0.0171 (14)	0.0038 (15)	0.0051 (14)
O1	0.0407 (15)	0.0597 (18)	0.0563 (19)	0.0306 (14)	0.0186 (14)	0.0269 (15)
O2	0.051 (2)	0.101 (3)	0.114 (3)	0.048 (2)	0.048 (2)	0.062 (3)
O3	0.0378 (14)	0.0530 (17)	0.0492 (18)	0.0261 (13)	0.0171 (13)	0.0252 (14)
O4	0.0361 (14)	0.0392 (15)	0.0574 (19)	0.0239 (12)	0.0182 (13)	0.0205 (13)
O5	0.0308 (13)	0.0289 (13)	0.066 (2)	0.0114 (12)	0.0099 (13)	0.0200 (13)
O6	0.0397 (17)	0.059 (2)	0.094 (3)	0.0287 (15)	0.0296 (17)	0.0359 (18)
O1W	0.235 (12)	0.129 (7)	0.141 (7)	0.008 (6)	0.044 (8)	0.040 (5)
O2W	0.065 (3)	0.095 (3)	0.243 (8)	0.049 (3)	0.060 (4)	0.094 (4)
O3W	0.090 (3)	0.208 (6)	0.083 (3)	0.101 (4)	0.045 (3)	0.068 (4)
O4W	0.130 (5)	0.110 (5)	0.187 (8)	0.020 (4)	0.073 (6)	0.018 (5)

### Geometric parameters (Å, °)

**Table:**

Cu1—O3	1.936 (3)	C18—C19	1.428 (5)
Cu1—O1	1.939 (3)	C19—O5	1.268 (5)
Cu1—O4	1.944 (3)	C19—C20	1.441 (5)
Cu1—O5	1.953 (3)	C20—C25	1.404 (5)
Cu1—N6^i^	2.248 (3)	C20—C21	1.405 (5)
C1—O2	1.229 (6)	C21—C22	1.352 (5)
C1—O1	1.285 (6)	C21—H21	0.9300
C1—C2	1.489 (6)	C22—F2	1.362 (4)
C2—C10	1.371 (6)	C22—C23	1.414 (6)
C2—C3	1.426 (6)	C23—C24	1.388 (6)
C3—O3	1.281 (5)	C23—N5	1.398 (5)
C3—C4	1.435 (6)	C24—C25	1.406 (5)
C4—C5	1.403 (6)	C24—H24	0.9300
C4—C9	1.406 (6)	C25—N4	1.398 (5)
C5—C6	1.344 (6)	C26—N4	1.340 (5)
C5—H5	0.9300	C26—H26	0.9300
C6—F1	1.357 (6)	C27—C28	1.511 (8)
C6—C7	1.420 (7)	C27—H27A	0.9600
C7—C8	1.388 (6)	C27—H27B	0.9600
C7—N2	1.384 (6)	C27—H27C	0.9600
C8—C9	1.396 (6)	C28—N4	1.483 (5)
C8—H8	0.9300	C28—H28A	0.9700
C9—N1	1.398 (5)	C28—H28B	0.9700
C10—N1	1.341 (6)	C29—N5	1.481 (6)
C10—H10	0.9300	C29—C30	1.505 (6)
C11—N1	1.475 (6)	C29—H29A	0.9700
C11—C12	1.499 (8)	C29—H29B	0.9700
C11—H11A	0.9700	C30—N6	1.481 (5)
C11—H11B	0.9700	C30—H30A	0.9700
C12—H12A	0.9600	C30—H30B	0.9700
C12—H12B	0.9600	C31—N6	1.464 (6)
C12—H12C	0.9600	C31—C32	1.513 (6)
C13—N2	1.488 (6)	C31—H31A	0.9700
C13—C14	1.517 (7)	C31—H31B	0.9700
C13—H13A	0.9700	C32—N5	1.458 (5)
C13—H13B	0.9700	C32—H32A	0.9700
C14—N3	1.452 (7)	C32—H32B	0.9700
C14—H14A	0.9700	N3—H3B	0.900 (8)
C14—H14B	0.9700	N6—Cu1^ii^	2.248 (3)
C15—N3	1.454 (7)	N6—H6A	1.05 (2)
C15—C16	1.511 (7)	O1W—O1W^iii^	1.500 (18)
C15—H15A	0.9700	O1W—H1W	0.83 (9)
C15—H15B	0.9700	O1W—H2W	0.83 (8)
C16—N2	1.469 (6)	O2W—H3W	0.83 (7)
C16—H16A	0.9700	O2W—H4W	0.83 (4)
C16—H16B	0.9700	O3W—H5W	0.88 (3)
C17—O6	1.236 (5)	O3W—H6W	0.83 (8)
C17—O4	1.270 (5)	O4W—H7W	0.83 (8)
C17—C18	1.503 (5)	O4W—H8W	0.90 (9)
C18—C26	1.369 (6)		
			
O3—Cu1—O1	92.22 (13)	C21—C20—C19	119.3 (3)
O3—Cu1—O4	85.49 (12)	C22—C21—C20	119.4 (3)
O1—Cu1—O4	164.06 (13)	C22—C21—H21	120.3
O3—Cu1—O5	165.39 (13)	C20—C21—H21	120.3
O1—Cu1—O5	87.15 (13)	C21—C22—F2	117.8 (3)
O4—Cu1—O5	91.12 (12)	C21—C22—C23	123.8 (3)
O3—Cu1—N6^i^	105.05 (13)	F2—C22—C23	118.4 (3)
O1—Cu1—N6^i^	94.09 (14)	C24—C23—N5	123.3 (3)
O4—Cu1—N6^i^	101.75 (14)	C24—C23—C22	116.5 (3)
O5—Cu1—N6^i^	89.55 (13)	N5—C23—C22	120.1 (3)
O2—C1—O1	122.4 (4)	C23—C24—C25	120.6 (4)
O2—C1—C2	118.5 (4)	C23—C24—H24	119.7
O1—C1—C2	119.1 (4)	C25—C24—H24	119.7
C10—C2—C3	118.6 (4)	N4—C25—C20	118.1 (3)
C10—C2—C1	116.4 (4)	N4—C25—C24	121.2 (3)
C3—C2—C1	125.1 (4)	C20—C25—C24	120.7 (3)
O3—C3—C2	125.0 (4)	N4—C26—C18	125.0 (4)
O3—C3—C4	118.1 (4)	N4—C26—H26	117.5
C2—C3—C4	117.0 (4)	C18—C26—H26	117.5
C5—C4—C9	117.8 (4)	C28—C27—H27A	109.5
C5—C4—C3	120.8 (4)	C28—C27—H27B	109.5
C9—C4—C3	121.4 (4)	H27A—C27—H27B	109.5
C6—C5—C4	120.4 (4)	C28—C27—H27C	109.5
C6—C5—H5	119.8	H27A—C27—H27C	109.5
C4—C5—H5	119.8	H27B—C27—H27C	109.5
C5—C6—F1	118.4 (4)	N4—C28—C27	111.2 (4)
C5—C6—C7	123.9 (4)	N4—C28—H28A	109.4
F1—C6—C7	117.6 (4)	C27—C28—H28A	109.4
C8—C7—N2	123.2 (4)	N4—C28—H28B	109.4
C8—C7—C6	115.3 (4)	C27—C28—H28B	109.4
N2—C7—C6	121.4 (4)	H28A—C28—H28B	108.0
C7—C8—C9	122.2 (4)	N5—C29—C30	110.5 (4)
C7—C8—H8	118.9	N5—C29—H29A	109.5
C9—C8—H8	118.9	C30—C29—H29A	109.6
C8—C9—N1	121.1 (4)	N5—C29—H29B	109.5
C8—C9—C4	120.3 (4)	C30—C29—H29B	109.5
N1—C9—C4	118.5 (4)	H29A—C29—H29B	108.1
N1—C10—C2	124.8 (4)	N6—C30—C29	110.2 (3)
N1—C10—H10	117.6	N6—C30—H30A	109.6
C2—C10—H10	117.6	C29—C30—H30A	109.6
N1—C11—C12	112.5 (4)	N6—C30—H30B	109.6
N1—C11—H11A	109.1	C29—C30—H30B	109.6
C12—C11—H11A	109.1	H30A—C30—H30B	108.1
N1—C11—H11B	109.1	N6—C31—C32	110.6 (4)
C12—C11—H11B	109.1	N6—C31—H31A	109.5
H11A—C11—H11B	107.8	C32—C31—H31A	109.5
C11—C12—H12A	109.5	N6—C31—H31B	109.5
C11—C12—H12B	109.5	C32—C31—H31B	109.5
H12A—C12—H12B	109.5	H31A—C31—H31B	108.1
C11—C12—H12C	109.5	N5—C32—C31	110.2 (3)
H12A—C12—H12C	109.5	N5—C32—H32A	109.6
H12B—C12—H12C	109.5	C31—C32—H32A	109.6
N2—C13—C14	110.1 (4)	N5—C32—H32B	109.6
N2—C13—H13A	109.6	C31—C32—H32B	109.6
C14—C13—H13A	109.6	H32A—C32—H32B	108.1
N2—C13—H13B	109.6	C10—N1—C9	119.6 (4)
C14—C13—H13B	109.6	C10—N1—C11	118.2 (4)
H13A—C13—H13B	108.1	C9—N1—C11	122.1 (4)
N3—C14—C13	113.2 (4)	C7—N2—C16	117.2 (4)
N3—C14—H14A	109.0	C7—N2—C13	116.2 (4)
C13—C14—H14A	109.0	C16—N2—C13	110.8 (4)
N3—C14—H14B	108.9	C14—N3—C15	109.1 (4)
C13—C14—H14B	108.9	C14—N3—H3B	124.2 (12)
H14A—C14—H14B	107.8	C15—N3—H3B	123.9 (12)
N3—C15—C16	114.5 (4)	C26—N4—C25	119.5 (3)
N3—C15—H15A	108.6	C26—N4—C28	117.4 (3)
C16—C15—H15A	108.6	C25—N4—C28	123.0 (3)
N3—C15—H15B	108.6	C23—N5—C32	117.2 (3)
C16—C15—H15B	108.6	C23—N5—C29	115.6 (3)
H15A—C15—H15B	107.6	C32—N5—C29	110.5 (3)
N2—C16—C15	110.3 (4)	C31—N6—C30	108.5 (3)
N2—C16—H16A	109.6	C31—N6—Cu1^ii^	115.4 (3)
C15—C16—H16A	109.6	C30—N6—Cu1^ii^	119.5 (3)
N2—C16—H16B	109.6	C31—N6—H6A	111.7 (13)
C15—C16—H16B	109.6	C30—N6—H6A	111.6 (13)
H16A—C16—H16B	108.1	Cu1^ii^—N6—H6A	89 (2)
O6—C17—O4	122.8 (4)	C1—O1—Cu1	131.2 (3)
O6—C17—C18	117.7 (4)	C3—O3—Cu1	127.2 (3)
O4—C17—C18	119.6 (3)	C17—O4—Cu1	130.5 (2)
C26—C18—C19	118.9 (3)	C19—O5—Cu1	126.1 (2)
C26—C18—C17	117.4 (3)	O1W^iii^—O1W—H1W	161 (7)
C19—C18—C17	123.7 (3)	O1W^iii^—O1W—H2W	85 (7)
O5—C19—C18	125.4 (3)	H1W—O1W—H2W	113 (10)
O5—C19—C20	118.8 (3)	H3W—O2W—H4W	113 (8)
C18—C19—C20	115.8 (3)	H5W—O3W—H6W	108 (9)
C25—C20—C21	118.4 (3)	H7W—O4W—H8W	108 (11)
C25—C20—C19	122.2 (3)		

Symmetry codes: (i) *x*, *y*−1, *z*; (ii) *x*, *y*+1, *z*; (iii) −*x*+1, −*y*+2, −*z*.

### Hydrogen-bond geometry (Å, °)

**Table:**

*D*—H···*A*	*D*—H	H···*A*	*D*···*A*	*D*—H···*A*
O1W—H1W···O4W	0.83 (9)	2.08 (10)	2.735 (14)	136 (10)
O1W—H2W···O3W^ii^	0.83 (8)	1.78 (8)	2.580 (11)	163 (11)
O3W—H5W···O1W^i^	0.88 (6)	2.17 (11)	2.580 (11)	108 (9)
O3W—H6W···O1	0.83 (8)	2.13 (8)	2.865 (8)	147 (9)
O4W—H8W···O1W	0.90 (9)	2.17 (11)	2.735 (14)	121 (10)
N6—H6A···O1W^iii^	1.05 (3)	2.12 (4)	3.105 (12)	155 (3)

Symmetry codes: (ii) *x*, *y*+1, *z*; (i) *x*, *y*−1, *z*; (iii) −*x*+1, −*y*+2, −*z*.
